# Correction: Brush-like block copolymer synthesized *via* RAFT polymerization for graphene oxide aqueous suspensions

**DOI:** 10.1039/d5ra90132d

**Published:** 2025-11-13

**Authors:** Min Qiao, Shishan Wu, Yanwei Wang, Qianping Ran

**Affiliations:** a School of Chemistry and Chemical Engineering, Nanjing University, Xianlin Campus 163 Xianlin Avenue, Qixia District Nanjing 210023 P. R. China shishanwu@nju.edu.cn; b State Key Laboratory of High Performance Civil Engineering Materials, Jiangsu Sobute New Material Co. Ltd 118 Liquan Road, Jiangning District Nanjing 211103 China

## Abstract

Correction for ‘Brush-like block copolymer synthesized *via* RAFT polymerization for graphene oxide aqueous suspensions’ by Min Qiao *et al.*, *RSC Adv.*, 2017, **7**, 4776–4782, https://doi.org/10.1039/C6RA27480C.

The authors regret that due to an inappropriate smoothing method used in the generation of Fig. 5 of the original article and the absence of the vertical axis in the figure, the XRD patterns for the three suspensions look very similar. A corrected Fig. 5 is provided here, using the original unsmoothed data with a vertical axis. The peak positions of the three samples are consistent with the description in the original text.

**Fig. 5:**
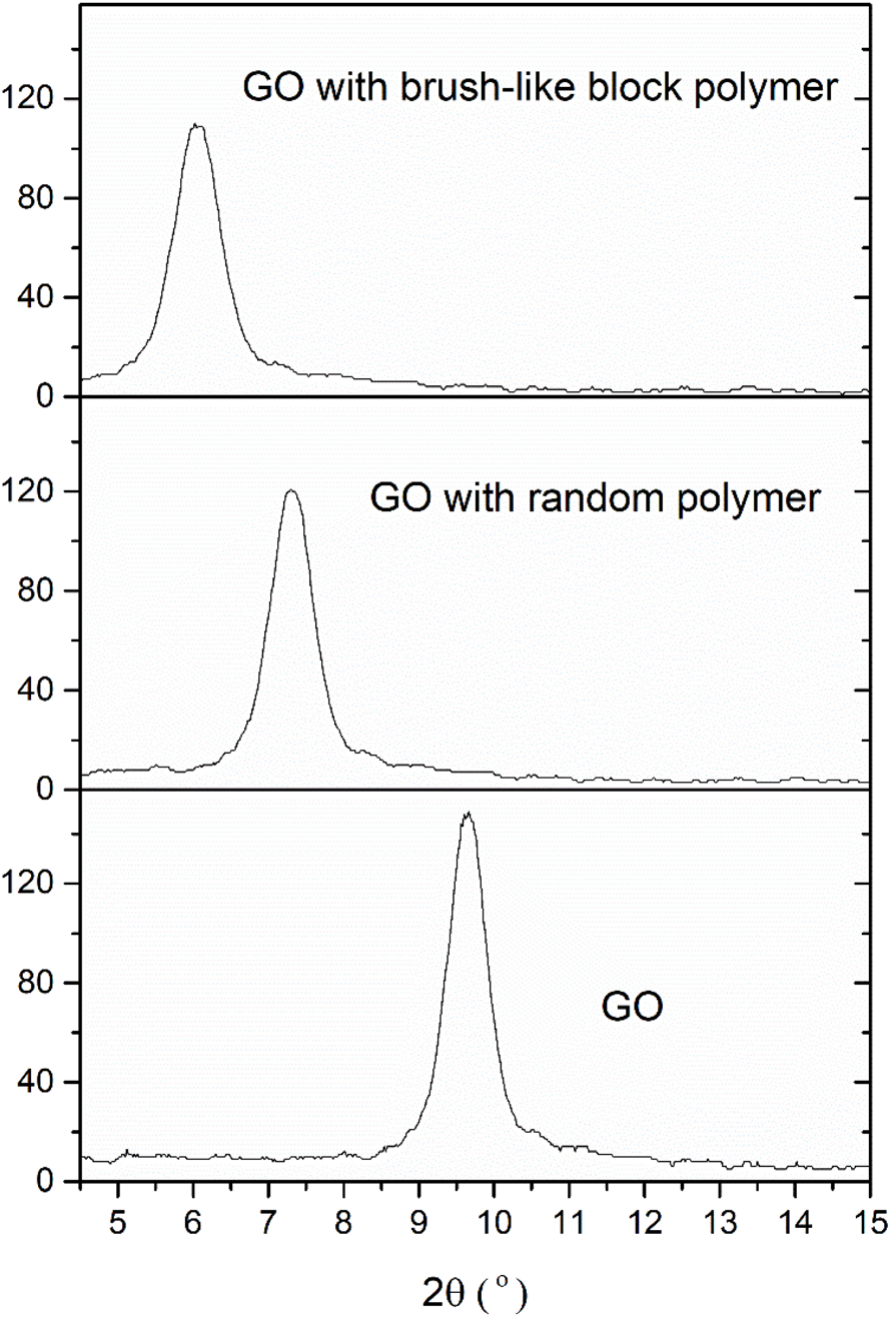
Comparison of the small angle XRD patterns for GO suspensions with and without dispersant.

The authors also found that in the process of creating Fig. 7, the data for 3 days was mistakenly reused as the data for 7 days. A corrected Fig. 7 with the correct 7-day data is shown. The conclusion shown in the corrected Fig. 7 is consistent with the description in the original published paper.

**Fig. 7:**
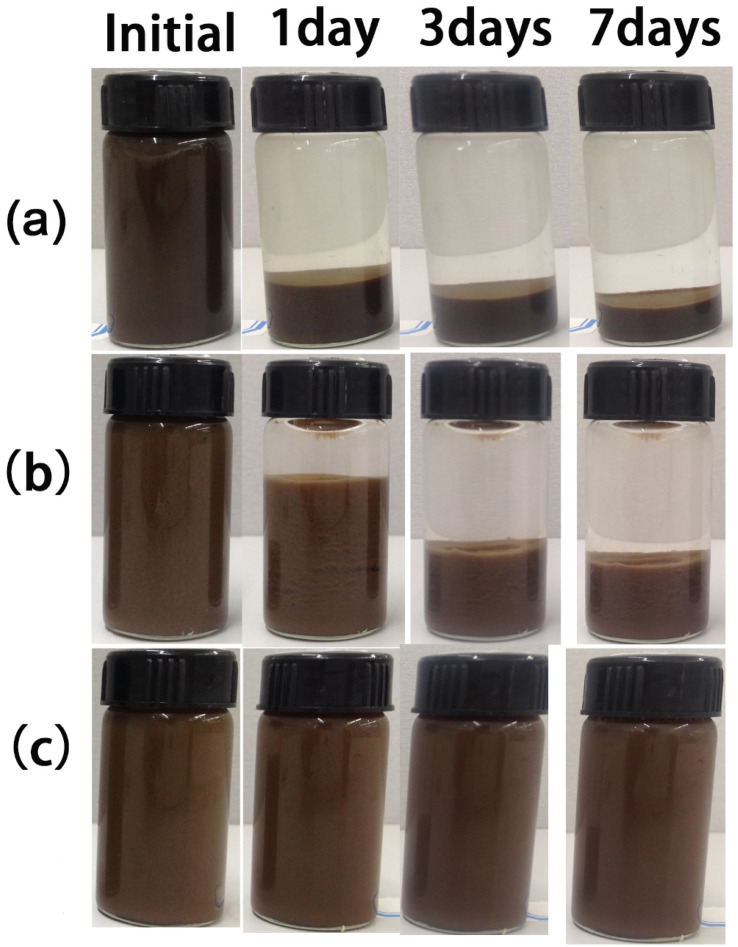
Photographs of GO aqueous suspensions at different storage time for samples without dispersant (a) and with the comparative random copolymer (b) or the prepared brush-like copolymer (c) as dispersant

An independent expert has viewed the corrected images and the raw data and has concluded that they are consistent with the discussions and conclusions presented.

The Royal Society of Chemistry apologises for these errors and any consequent inconvenience to authors and readers.

